# Navigating Ethics in the Digital Age: Introducing Connected and Open Research Ethics (CORE), a Tool for Researchers and Institutional Review Boards

**DOI:** 10.2196/jmir.6793

**Published:** 2017-02-08

**Authors:** John Torous, Camille Nebeker

**Affiliations:** ^1^ Department of Psychiatry Beth Israel Deaconess Medical Center Harvard Medical School Boston, MA United States; ^2^ Division of Clinical Informatics Beth Israel Deaconess Medical Center Harvard Medical School Boston, MA United States; ^3^ Department of Family Medicine and Public Health Division of Behavioral Medicine UC San Diego School of Medicine La Jolla, CA United States; ^4^ Center for Wireless and Population Health Systems Qualcomm Institute UC San Diego La Jolla, CA United States; ^5^ Scripps Translational Science Institute La Jolla, CA United States

**Keywords:** ethics, research, mHealth, research, mobile technologies, telemedicine

## Abstract

Research studies that leverage emerging technologies, such as passive sensing devices and mobile apps, have demonstrated encouraging potential with respect to favorably influencing the human condition. As a result, the nascent fields of mHealth and digital medicine have gained traction over the past decade as demonstrated in the United States by increased federal funding for research that cuts across a broad spectrum of health conditions. The existence of mHealth and digital medicine also introduced new ethical and regulatory challenges that both institutional review boards (IRBs) and researchers are struggling to navigate. In response, the Connected and Open Research Ethics (CORE) initiative was launched. The CORE initiative has employed a participatory research approach, whereby researchers and IRB affiliates are involved in identifying the priorities and functionality of a shared resource. The overarching goal of CORE is to develop dynamic and relevant ethical practices to guide mHealth and digital medicine research. In this Viewpoint paper, we describe the CORE initiative and call for readers to join the CORE Network and contribute to the bigger conversation on ethics in the digital age.

## Introduction

The transformative potential of mobile, digital, and passive sensing technologies to observe or intervene with various health domains is now well established. As the field transitions from discussing to now testing this potential, the role of clinical studies and evidence generation will assume predominance.

While there are many new challenges in conducting technology-enhanced research, even navigating study approval via the institutional review board (IRB) ethical and regulatory evaluation process remains a primary challenge for many. Whether the existing IRB system is able to support the ethical review and subsequent advancement of research using these new and emerging technologies was recently questioned [1]. To address ethical challenges in this new age of technology-enhanced research, we outline a potential solution.

Health-related research using tools, such as social networks, mobile phones apps, and wearable passive sensors, offer the potential to collect unprecedented amounts of real-time data outside of the clinic and in real-world or “free-living” environments. From monitoring location using a mobile phone’s global positioning sensors to inferring socialness based on call and text message logs, mobile and passive sensing technologies offer a new window into human behavior. Yet such broad data capture can also have numerous unintended consequences [2,3]. Research participants may be unaware of the nature, scope, and granularity of data collected and what information they are actually consenting to provide. Wearable recording may capture the sounds and images of nearby people not involved in the research, or “bystanders,” due to their close proximity to a research participant [4]. Text message logs may also reveal personal communications from and with bystanders, introducing potential privacy concerns and legal challenges [5]. It is easy to imagine many more examples where both personal and community privacy boundaries can easily be infringed upon and compromised.

Many researchers are eager to study the clinical potential of mobile and digital tools, but they may not be aware of all the potential risks to participants, or of means to mitigate them [6,7]. IRBs have thus been placed in the difficult role of evaluating these research proposals and ensuring they are both safe and ethical. Yet, like many researchers, IRB members themselves are not experts in this emerging field and may struggle to evaluate the safety and ethical dimensions of submissions that often involve novel uses of various technologies. In many cases, there are actually no standards, best practices, or demonstrated safety mechanisms to guide either researchers or IRB risk assessment or management strategies. Researchers may thus feel uncomfortable with explaining digital or mHealth benefits, risks, and management strategies in the IRB protocol application and, likewise, IRBs may feel uncomfortable approving study plans. The outcome is a lengthy IRB review process that may result in either the underprotection or overprotection of research participants [3,4].

Given the evolving state of knowledge and growing interest in using technologies, one simple solution is sharing of developing practices and examples of successful digital- and mHealth-related IRB submissions. The idea of sharing resources to guide the ethical conduct of research using novel strategies is not new. In fact, sharing guidance was initiated years ago when researchers started to use the Internet to support scientific inquiry. For example, the Association of Internet Research is a membership organization that aims to foster ethical and professional Internet research by offering guidance and education to academics, including faculty and students [8]. Similarly, the British Psychological Society has developed ethical guidance for Internet-mediated research [9], as has INVOLVE [10], which is a Web-based resource launched in the United Kingdom 20 years ago with support from the National Institute for Health Research. While these resources focus primarily on Internet research, there are other organizations thinking about ethical dimensions of nanoengineering, robotics, and artificial intelligence, yet not necessarily focusing on research with human participants. Several years ago, we recognized that researchers and ethics review boards (eg, IRBs or research ethics boards) may benefit from having access to a community of stakeholders with expertise in various digital and mHealth tools. The initial goal was to create an accessible and dynamic resource to bridge a growing gap between researchers who capture personal health data via mobile (apps), imaging (eg, Microsoft’s SenseCam wearable camera), pervasive sensing (eg, wearable, ingestible, and environmental technology), social media (eg, Twitter, Instagram, and Facebook), and geolocation tracking (eg, global positioning systems and geographic information systems) tools and the IRBs charged with reviewing these studies.

## Introducing the Connected and Open Research Ethics Initiative

The Connected and Open Research Ethics (CORE) initiative was launched in 2015 with support from the Robert Wood Johnson Foundation (Princeton, NJ). The CORE is a free, Web-based resource that aims to convene stakeholders in the digital-mHealth ecosystem to collectively shape dynamic and responsive ethical and responsible research practices. Using a participatory approach to inform the CORE design and function, the CORE team invited input from individuals representing interdisciplinary, cross-disciplinary, and cross-sector perspectives with a vested interest in advancing dynamic and responsive ethical standards. Focus groups and key informant interviews were convened with IRB representatives and researcher stakeholders to inform the initial CORE Platform functionality and design. The CORE Platform, released in 2016, hosts a growing global network of over 200 individuals representing 10 countries and a majority of the United States with expertise in privacy, technology, bioethics, research ethics, regulations, sciences, engineering, and even a few participants. The key features include a Forum where Network members can share informational resources and post or answer questions, and a Resource Library where researchers can upload language used in their IRB-approved protocol application and informed consent documents. The goal is to help other researchers who are beginning to use new digital tools in research and who want to see examples of successful IRB protocol and consent language, and receive feedback from experts when writing their own IRB applications.

**Figure 1:**
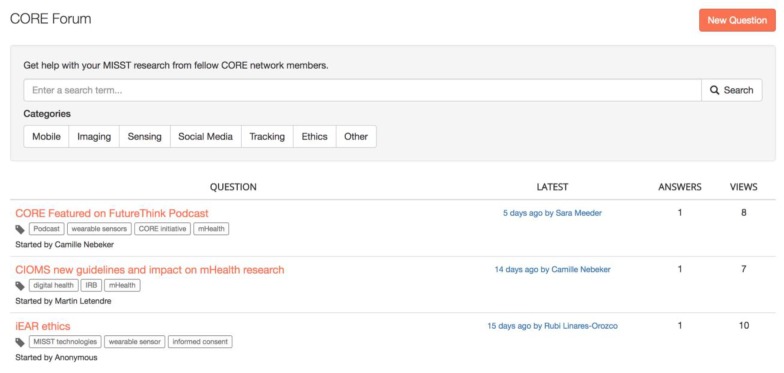
Connected and Open Research Ethics (CORE) Forum screenshot. MISST: mobile imaging, pervasive sensing, social media and location tracking.

**Figure 2:**
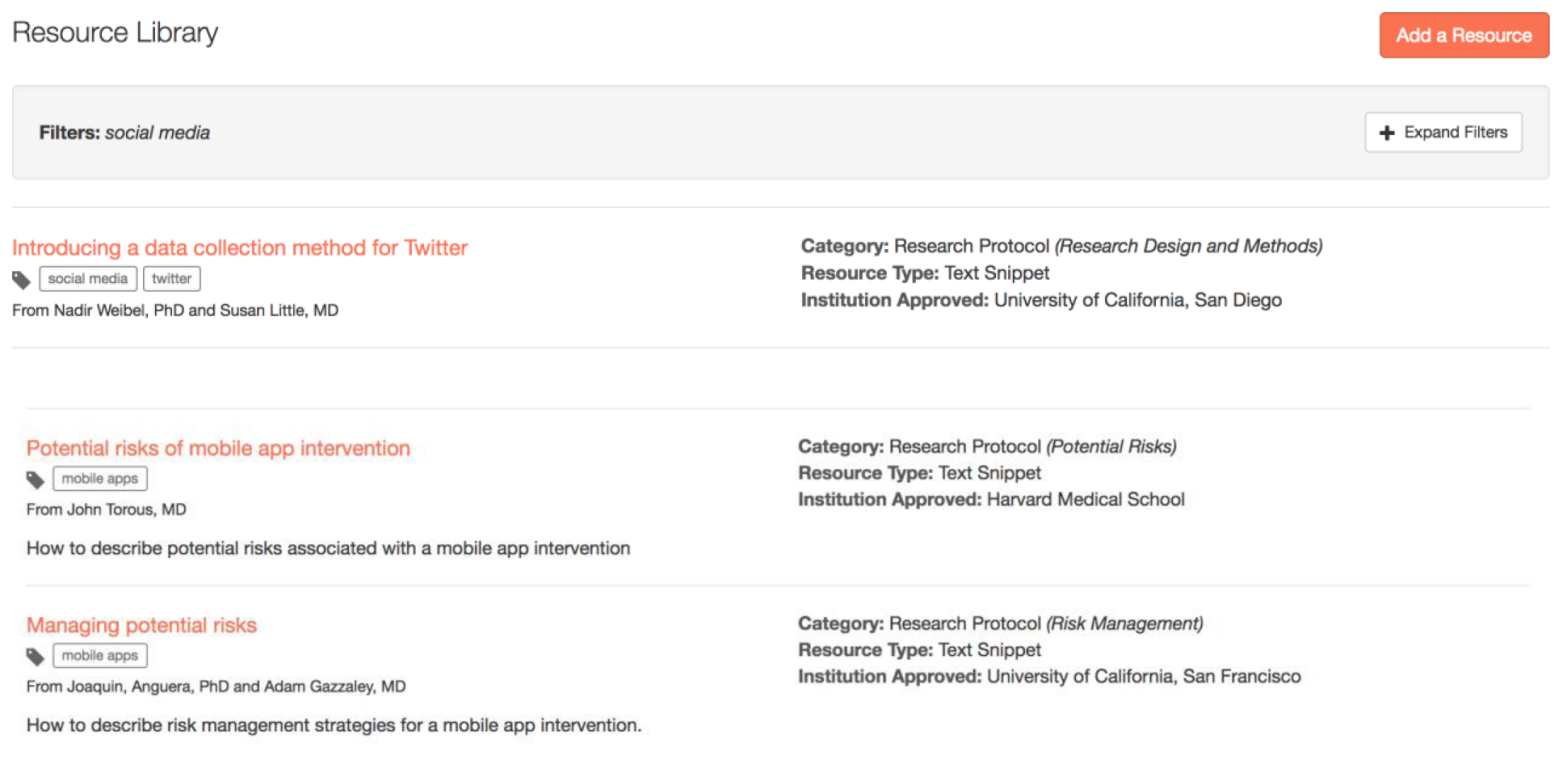
Connected and Open Research Ethics (CORE) Resource Library screenshot.

Likewise, IRBs that are beginning to review mHealth and digital medicine research studies can post questions on the CORE Forum (Figure 1), as well as contribute to or search the Resource Library (Figure 2), to see what others have found to be acceptable. This saves time and, ideally, increases the consistency for how IRBs evaluate and mitigate potential risks to research participants.

That being said, we cannot be certain that an IRB approval means that the ethical review is beyond reproach. As the CORE community begins to share resources, we expect stakeholders to chime in when a potential risk has been overlooked or, likewise, when the approved protocol appears overly conservative. The CORE is also where new resources (eg, institutional policies or guidelines) can be shared and ideas explored, which may lead to potential collaborations.

As with any innovation, early adopters to the CORE also serve as beta testers who help to improve utility and functionality. As we enter this new frontier where vast and granular amounts of personal health data are collected in real time and 24/7, we look to the CORE Network members to begin shaping how to do this research in a manner that is informed, ethical, and responsive to participants’ interests. The CORE Resource Library is designed so that as tools or methods become obsolete, they will drop off and the new innovations will percolate to the top. Moreover, the CORE has a rating system so that if a Network member notes a gap in the data security plan, they can chime in and inform the community of a better practice.

We invite readers of the *Journal of Medical Internet Research* to join the CORE Network to share knowledge, access resources, and contribute to shaping the ethics for 21st century research. Get started by visiting CORE’s website [11] and sign in to create an account. Once you are on the CORE Platform, browse the Resource Library and visit the Forum to engage in discussion with others in the CORE community.

## Abbreviations

CORE: Connected and Open Research Ethics
IRB: institutional review board

## References

[1] Bloss C, Nebeker C, Bietz M, Bae D, Bigby B, Devereaux M, Fowler J, Waldo A, Weibel N, Patrick K, Klemmer S, Melichar L (2016). Reimagining human research protections for 21st century science. J Med Internet Res.

[2] Torous J, Keshavan M, Gutheil T (2014). Promise and perils of digital psychiatry. Asian J Psychiatr.

[3] Huckvale K, Prieto JT, Tilney M, Benghozi P, Car J (2015). Unaddressed privacy risks in accredited health and wellness apps: a cross-sectional systematic assessment. BMC Med.

[4] Nebeker C, Lagare T, Takemoto M, Lewars B, Crist K, Bloss CS, Kerr J (2016). Engaging research participants to inform the ethical conduct of mobile imaging, pervasive sensing, and location tracking research. Transl Behav Med.

[5] Nebeker C, Linares-Orozco R, Crist K (2015). A multi-case study of research using mobile imaging, sensing and tracking technologies to objectively measure behaviorthical issues and insights to guide responsible research practice. J Res Admin.

[6] Pisani AR, Wyman PA, Mohr DC, Perrino T, Gallo C, Villamar J, Kendziora K, Howe GW, Sloboda Z, Brown CH (2016). Human subjects protection and technology in prevention science: selected opportunities and challenges. Prev Sci.

[7] Torous J, Roberts LW (2017). The ethical use of mobile health technology in clinical psychiatry. J Nerv Ment Dis.

[8] (2017). Association of Internet Researchers.

[9] Hewson C, Buchanan T, Brown I, Coulson N, Hagger-Johnson G, Joinson A, Krotoski A, Oates J (2013). Ethics guidelines for Internet-mediated research.

[10] National Institute for Health Research (2015). INVOLVE.

[11] (2016). CORE Platform.

